# Rapid Titration of Measles and Other Viruses: Optimization with Determination of Replication Cycle Length

**DOI:** 10.1371/journal.pone.0024135

**Published:** 2011-09-07

**Authors:** Boyan Grigorov, Jessica Rabilloud, Philip Lawrence, Denis Gerlier

**Affiliations:** INSERM, U758, Ecole Normale Supérieure de Lyon, Lyon, France, Université de Lyon, Lyon, France; University of Texas HSC - San Antonio, United States of America

## Abstract

**Background:**

Measles virus (MV) is a member of the *Paramyxoviridae* family and an important human pathogen causing strong immunosuppression in affected individuals and a considerable number of deaths worldwide. Currently, measles is a re-emerging disease in developed countries. MV is usually quantified in infectious units as determined by limiting dilution and counting of plaque forming unit either directly (PFU method) or indirectly from random distribution in microwells (TCID50 method). Both methods are time-consuming (up to several days), cumbersome and, in the case of the PFU assay, possibly operator dependent.

**Methods/Findings:**

A rapid, optimized, accurate, and reliable technique for titration of measles virus was developed based on the detection of virus infected cells by flow cytometry, single round of infection and titer calculation according to the Poisson's law. The kinetics follow up of the number of infected cells after infection with serial dilutions of a virus allowed estimation of the duration of the replication cycle, and consequently, the optimal infection time. The assay was set up to quantify measles virus, vesicular stomatitis virus (VSV), and human immunodeficiency virus type 1 (HIV-1) using antibody labeling of viral glycoprotein, virus encoded fluorescent reporter protein and an inducible fluorescent-reporter cell line, respectively.

**Conclusion:**

Overall, performing the assay takes only 24–30 hours for MV strains, 12 hours for VSV, and 52 hours for HIV-1. The step-by-step procedure we have set up can be, in principle, applicable to accurately quantify any virus including lentiviral vectors, provided that a virus encoded gene product can be detected by flow cytometry.

## Introduction

**Figure 1:**
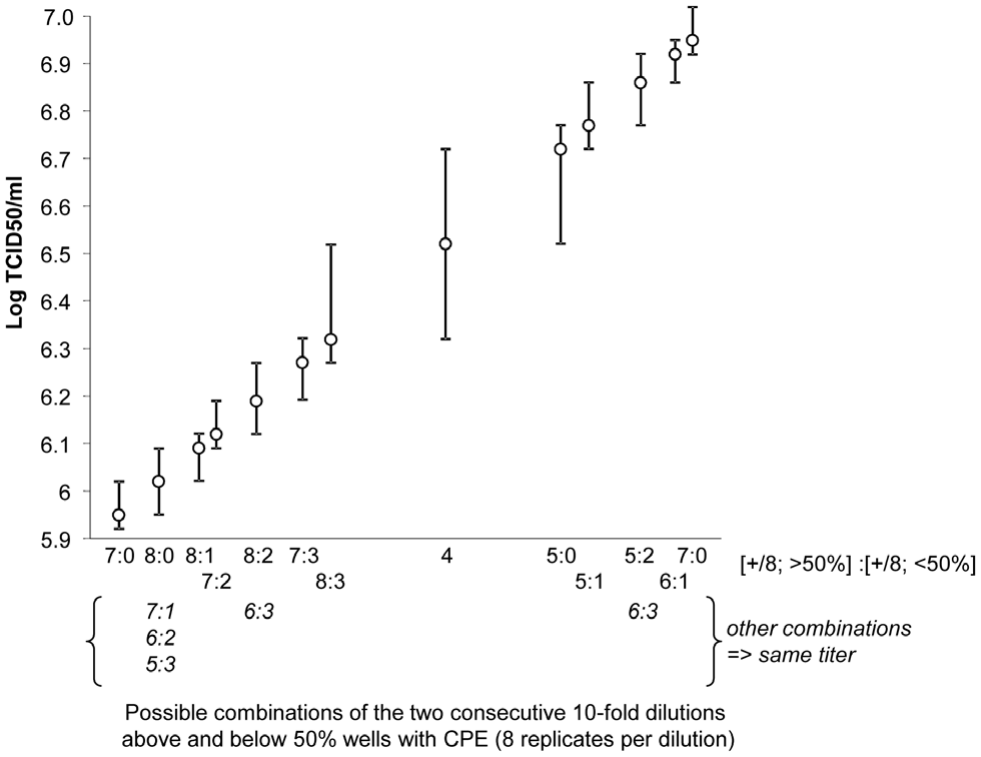
Virus titration by TCID50 is intrinsically discontinuous. Discontinuous titer values given by all possible combinations of percentage of infected wells above, at, and below 50% of eight replicates within one Log range. Note that (i) some mantissa are identical for two or four combinations (indicated in italics in x axis) and (ii) identical sets of mantissa of the logarithm are obtained at any place below and above the displayed titer range. Vertical bars indicate the variable interval separating one titer value from its nearest superior and inferior titer value.

Determining the amount of infectious virus is a crucial issue for any virologist. To date, different methods have been used for viral titration depending on the virus concerned. The most popular ones are the plaque forming units (PFU) [1] and the 50% tissue culture infective dose (TCID50) [2]. They are used for cytopathic viruses (e.g. HIV-1, poliovirus, Japanese encephalitis virus, measles virus, etc…) and are based on serial dilutions of the virus-containing samples and observation of the appearance of a cytopathic effect (CPE) in a cell monolayer. The PFU technique measures the number of virus particles capable of forming plaques per volume unit, and accounts for the replication potency of the virions, i.e. it is a functional measure. The PFU assay is laborious, poorly automatable, and requires several hours to several days according to the speed of virus growth and propagation. It also suffers from subjectivity because of the tedious manual plaque counting and possible plaque-like defects in the cell monolayer. The TCID50 method is a statistical derivative of the PFU assay. Instead of counting individual plaques, multiple replicates of each virus dilution are made and the TCID50 titer is calculated from the 50% endpoint where half of the replicates contained at least one PFU. Wells with destroyed cell monolayers are easily counted either manually under the microscope or automatically using a viable colorimetric assay. TCID50 values are intrinsically discontinuous, and the value coverage is non-homogeneous (i.e. intervals between two discontinuous values are not identical) as illustrated in Figure 1. Therefore, increasing the measurement accuracy would require a number of replicates high enough to discourage routine usage. Handling and time requirements of TCID50 and PFU methods are comparable. With both assays, virus titration is more difficult with cells growing in suspension.

Due to the inconveniences mentioned above for these two titration methods, novel, quicker and less cumbersome techniques have been developed. Such titration procedures have been developed for influenza viruses, adenoviruses, HIV-1, SV40, human coronaviruses, hepatitis A virus, as well as for recombinant and/or virus-like particles [3]–[13]. Many of these techniques are based on the identification of infected cells at the single cell level using immunodetection of viral proteins (structural or glycoproteins) by flow cytometry [9],[7],[13]–[14]. In such studies, the virus titer is calculated from the proportion of infected cells (i.e. positively labeled) after exposure of a given number of indicator cells to a given virus suspension volume. In each case, the assumption is made that one cell is infected by a single infectious virus particle. However, at a too high multiplicity of infection (MOI) (i.e. the ratio of infectious virus particle per cell) such an assumption becomes erroneous, leading to an underestimation of virus titers. Indeed, the higher the MOI, the higher the probability of multiple viruses infecting a single cell. Likewise, secondary infections of cells by newly produced viruses during the assay lead to overestimation of the virus titer.

To avoid these pitfalls, we have explored the prerequisites and limitations of the use of flow cytometry for virus quantification with a specific focus on measles virus (MV) strains, while taking into account the following parameters: (i) virus infected cells must be clearly distinguished from the uninfected ones by emitting a fluorescent signal above the autofluorescence background; (ii) the virus titer is best determined by averaging titers calculated from several amounts of the viral inoculum; (iii) the measurement should be optimally done at, or just prior to, the completion of the first viral replication cycle to avoid secondary infection; (iv) virus titer should be calculated from the number of uninfected cells by applying the Poisson probability distribution and (v) the method should be validated by comparison with an already existing titration technique e.g. TCID50. In addition, the assay could be used to approximate the duration of a single replication cycle. Our method was validated by the strong correlation found with the TCID50 technique over a large set of samples. The assay lasts less than two days which favorably compares with the ∼1–2 week duration of the conventional PFU or TCID50 methods currently used for MV titration.

In order to validate this rapid assay for measles virus titration, we have compared it to the classical TCID50 method. A high amount of virus samples quantified by both techniques showed a strong correlation indicating thus the accuracy of the novel method. The detection limit is 10^4^ infectious units/ml.

To illustrate its wide application, our method was also adapted to titrate recombinant MV and vesicular stomatitis virus (VSV) expressing the green fluorescent protein (GFP) as gene reporter of the infection. Likewise, the method was also applicable to measure HIV-1 titer using a reporter cell line that conditionally expresses GFP under the control of the Tat-inducible HIV-1 LTR promoter. In the latter case, titer accuracy required the blocking of cell growth by the DNA polymerase inhibitor aphidicolin over the 2-day measurement period.

## Materials and Methods

### MV titration by TCID50

We have used TCID50 titration method as a reference for the validation of our rapid titration technique. TCID50 is based on the end-point dilution of the virus at which a cytopathic effect (CPE) is detected in 50% of the cell culture replicates infected by a given amount of virus suspension [2]. The latter was serially diluted in 0.25 ml of DMEM medium supplemented with 6% fetal calf serum (FCS) and antibiotics (culture medium) by serial transfer of 0.027 ml (i.e. ten-fold dilution) in the first row of a 96 well plate. When diluting the virus suspension, each micropipette tip ought to be changed at every dilution to avoid uneven distribution of infectious particles that remain adsorbed on the tip. Subsequently, 0.030 ml of each dilution was distributed vertically, i.e. 8 replicates for each dilution. Permissive cells, i.e. Vero cells, (10,000 cells in 0.2 ml) were seeded in each well, and incubated at 37°C in 5% C0_2_ and humid atmosphere. CPE induced by measles virus could be observed under the microscope after 3–4 days, but because CPE located in the well edge could hardly be spotted, the final counting of wells with CPE was done after 10 days. This incubation time can be much shorter for quicker growing viruses (i.e. 1–2 days for VSV). To avoid underestimation of virus titer, the incubation time should be adapted to the virus species and physicochemical conditions used for virus propagation. TCID50 was then calculated using the formula:
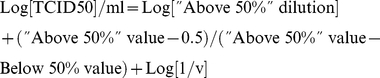
where “Above 50%” dilution is the last dilution expressed as the denominator of 1 (i.e. 250 for 1/250 dilution) for which ≥50% wells display CPE, (“Above 50%” and “Below 50%” values are fraction of wells with CPE at the last dilution for which ≥50% wells display CPE and fraction of wells with CPE at the next dilution, respectively, and v the virus volume (in ml) from the undiluted sample used to inoculate the wells.

### Step-by-step MV titration procedure by flow cytometry (protocol at a glance)

The flow cytometry titration method, based on the immunodetection of the viral F-glycoprotein on the surface of infected cells, was performed as shown below (an example of an assay timing is indicated when taking into account the following parameters: (i) titration of ∼20 samples of an MV strain with (ii) a replication cycle of 18 hours, (iii) without interruption of the assay until the calculation of the titer is done using the online spreadsheet).

#### 1. Start - cell seeding (10h00)

Seed 100 000 of Vero cells for MV laboratory/vaccine strains or Vero-Slam cells for MV - wild type strains in 0.25 ml of culture medium in each well of a 48 well plate. Preferably, plate the cells in the morning and infect them in the afternoon. Alternatively, inoculate the cells just after seeding. In that case perform the seed and inoculation in the afternoon. Do not seed the cells the day prior to the inoculation because of resulting change in cell number due to cell growth. The cell number seeded should be carefully adjusted since it is part of the formulae to calculate the virus titer in IU/ml (see below). Likewise, during the assay, cell growth should be very limited hence cell plating and infection on the same day. Indeed any overgrowth of uninfected cells versus infected cells during the titer assay will result in the underestimation of the virus titer (see also the application for titration of HIV-1 below).

#### 2. Virus inoculation (16h00)

**Figure 2:**
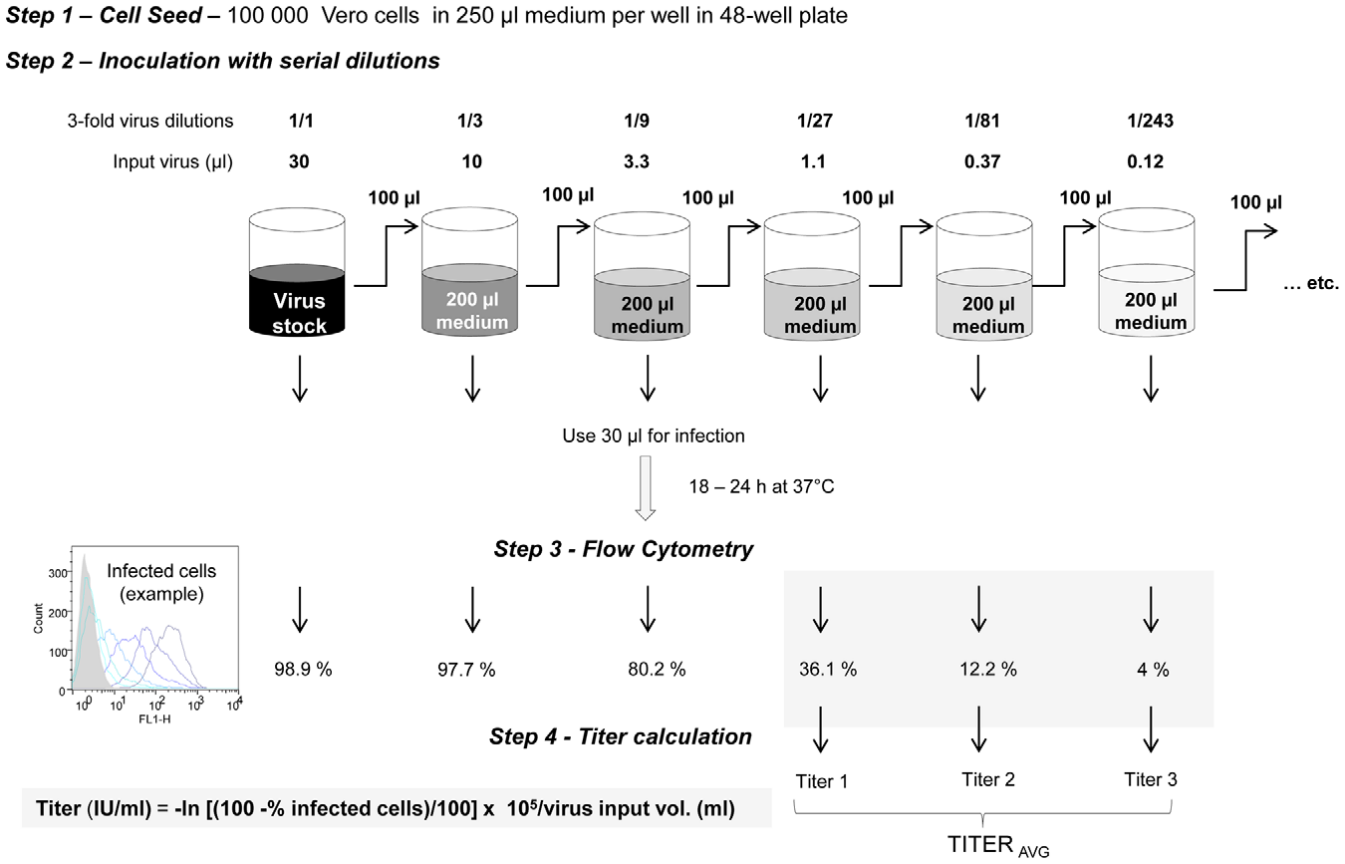
Experimental scheme and data analysis for virus titration based on flow cytometry numbering of infected cells – “Protocol at a glance”. Top panel from right to left, 3-fold dilution of unknown virus sample. Middle panel, example of histogram profiles of infected cells according to the virus inoculum size. Bottom panel: % of infected cells as graphically determined and equation used to calculate the titer for each inoculum volume. Underlined in grey, selected values for titer determination by averaging.

Prepare and add three-fold dilutions of the virus suspension to the cells in duplicate (see Figure 2 for a “protocol at a glance”). Fix the inoculation volume (virus+medium) to 0.03 ml. If lower titers are expected, increase the inoculation volume up to 0.090 or 0.27 ml of undiluted virus with complete replacement of the cell supernatant (0.25 ml) to avoid volume enlargement.

#### 3. Place the cells into an incubator at 37°C with 5% of CO_2_ supply (17h00)

Do not replace the culture cell medium.

#### 4. Flow cytometry: labeling (Day+1 10h00; time = 24 h)

*Option 1 - for MV vaccine and laboratory strains (i.e. Schwarz, Edmonston, Halle).*

- At 18 h post inoculation (next morning), discard the supernatant;
- Add 0.07 ml of trypsin to each well;
- Incubate for ∼5 minutes;
- add 0.2 ml of “wash medium” - DMEM supplemented with 6% FCS and 0.05% NaN_3_ (this medium blocks the trypsin and prevents any internalization of cell surface proteins, and should be used throughout the labeling procedure);
- Detach the cells by pipetting and individually transfer the cell suspensions into a V-bottom (or U-bottom) 96 well plate (when only a small number of samples and serial dilutions are carried on the procedure can be done in 1.5 ml conical tubes);
- Shortly centrifuge (3–5 min, 400×g, 4°C) the plate, carefully discard the supernatant to avoid any loss of cell pellet;
- Add 0.05 ml of anti-F monoclonal antibody optimally diluted in “wash medium” and resuspend the cells by pipetting;
- Incubate the plate for 30 min at 4°C;
- Wash the cells once by adding 0.25 ml of medium and pellet the cells by centrifugation (5 min, 400×g, 4°C).
- Add 0.05 ml of appropriately diluted in “wash medium” secondary fluorescent (PE or FITC) anti-mouse antibody and resuspend the cells by pipetting;
- Incubate the plate for 30 min at 4°C;
- Wash the cells once by adding 0.25 ml of medium and pellet the cells by centrifugation (5 min, 400×g, 4°C).
- Add 0.05 ml of 1% paraformaldehyde solution to the cell pellets, resuspend them by pipetting and leave for 5–10 min to allow cell fixation and virus inactivation;
- Add 0.3 ml of PBS.

*Day+1 12h00 (lunch break 12h00–14h00)*

- At this stage, the cells could be kept for 1 or 2 days at 4°C in the dark prior to the flow cytometry analysis.

*! Note that controls should include uninfected cells that have been treated according to the abovementioned procedure!*

*Option 2 – for MV wild type strains.* Perform cytometry 24 hours post inoculation following the same steps as described in “option 1”.

*Option 3 – for MV GFP encoding viruses.* Perform all the steps described in “option 1” except primary and secondary antibody labeling. After trypsinization, fix cells and perform the flow cytometry analysis.

#### 5. Flow cytometer acquisition (Day+1 14h00)

Set the side scatter (SSC) and the forward scatter (FSC) to select viable cells on unlabeled cell sample. Adjust the fluorescence background of the unlabeled cell sample to the lowest intensity (i.e. to have the fluorescent histogram close to the ordinate). Set the fluorescent threshold for positivity by gating more than 99.5% of the uninfected control cells outside the gate. Analyze every virus dilution by flow cytometry.

#### 6. Titer calculation (End of the titration assay Day+1 16h00)

First, determine the number of non-infected cells after infection, and then calculate for each sample the number of non-infected cells (N[n.i.]) within the 100 000 cell input according to the following
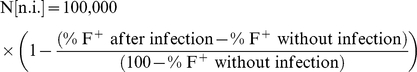
where F^+^ cells are cells with fluorescence above the >99.5% threshold defined above.

*Example:* For a threshold set to 0.3 %F^+^ on the uninfected control sample, and a percentage of infected cells %F^+^ = 10.3% in a test inoculated sample, then the number of non-infected after virus inoculation is
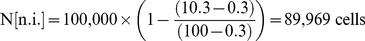
Calculate the titer from the number of uninfected cells: this titer estimation is based on the fact that the probability P(n) that one cell could be infected by “n” viral particles follows the Poisson law of the parameter “λ” which is equal to the multiplicity of infection (MOI).
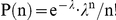
The probability that one cell will not be infected is then equal to **P(0) = e^−λ^**.

P(0) could be calculated experimentally by determining the proportion of uninfected cells from the total number of cells:
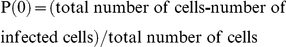
Consequently, the MOI (λ) = −ln P(0).

Calculate the titer using the formula:
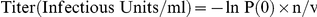
where P(0) is the fraction of uninfected cells (N[n.i.]/100 000), “*n*” is the number of cells in each well at the time of infection (i.e. 100 000) and “v” the undiluted virus input volume (in ml).

The Poisson's law is valid provided that the total number of cells is higher than 10, the number of infectious virus is higher than 30 and the MOI is below 10. While the two former parameters are always respected because of the high cell number and minimal virus input, MOI could be well over 10 for virus-rich samples. When the percentage of infected cells exceeds 30–40% the titer value tends to be underestimated. Thus, exclude values >40% infected cells from the calculation Likewise, exclude values below ∼0.5% infected cells since they fell too close to the background limit to be taken into account. Average the titers calculated from consecutive 3-fold dilution ranges showing ∼3-fold intervals.

*For automatic titer calculation, an online spreadsheet (csv file) is provided where the results from the flow cytometry for all viral inputs are simply entered where indicated. This file can be downloaded by following the link below (Ctrl+click):*

https://spreadsheets.google.com/ccc?key=0AvRhxJMlLBAAdHVaUk1fMDktOXZYQnA3dUo4UEgwV3c&hl=en

*You can also copy and paste the link into your browser.*

*Then, go to “File”→download→Excel*

### Materials

*Cells:* Vero -ATCC CCL-81; Vero-SLAM [15]; GHOST Cell Transfectants - GHOST (3) CXCR4 (NIH AIDS reagent program). *Media and reagents:* Dulbecco's Modified Eagle medium (DMEM); 0,05% Trypsine-EDTA; Phosphate Buffered Saline pH 7.2–7.4; Fetal Calf Serum; Measles virus anti F monoclonal antibody Y503 [16]–[17] (available upon request to D. Gerlier); Goat F(ab′)2 Fragment Anti-Mouse IgG (H+L)-FITC – Beckman Culter; Goat F(ab′)2 Fragment Anti-Mouse IgG (H+L)-PE – Beckman Culter; Sodium azide, Sigma-Aldrich; Paraformaldehyde 95% Powder, Sigma-Aldrich. *Plastics:* Becton Dickinson Falcon® Multiwell ™ 48 well; Nunc™ 96 well polystyrene, clear, V-bottom plates, VWR; Becton Falcon® 5 ml polystyrene round-bottom tubes 12×75 mm. *Equipments:* Flow cytometer; Refrigerating centrifuge for plates; Multichannel pipette (30–300 µl); Pipetman (1–20 µl, 20–200 µl, 100–1000 µl); Pipetman tips (1–200 and 100–1000 µl).

## Results and Discussion

### Comparison of the rapid method for MV titration with TCID50

**Figure 3:**
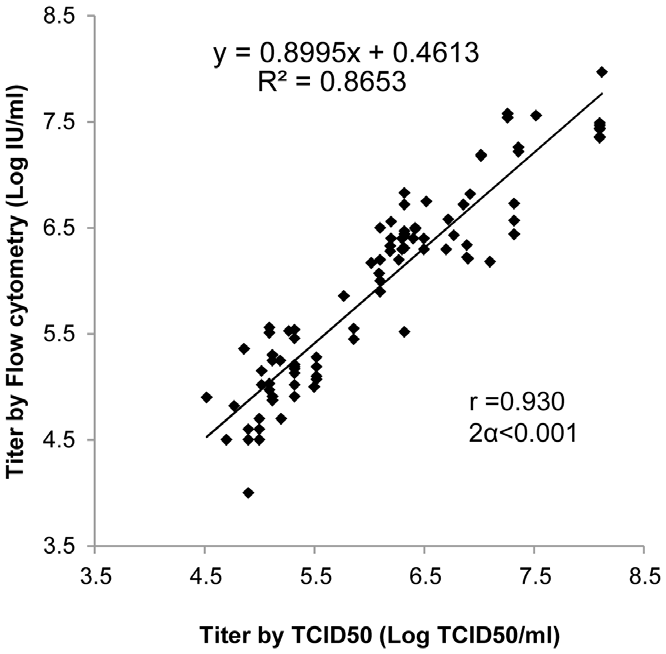
Correlation between titers obtained by the TCID50 technique and the rapid method for titration based on flow cytometry. Ninety five viral stocks from MV strains were titrated by the two techniques with highly correlated values (r = 0,930, 2α<0.001).

In order to validate the rapid protocol for measles virus titration, we have compared it to the classical TCID50 method. The latter was chosen instead of the PFU method since it is less laborious and operator dependent, there are no plaque-counting difficulties [18], and it has been routinely used by our laboratory over 20 years after internal validation in agreement with a large-scale European assessment [19] and WHO recommendations (http://www.who.int/vaccines-documents/DocsPDF-IBI-e/mod7_e.pdf). Six independent titrations of a given viral stock of MV-Schwarz strain gave a similar titer with 1% variation compared to 1.5% using the TCID50 titration method for the same sample. This demonstrates that the flow cytometry-based titration method is reproducible. Furthermore, titers of near one hundred MV stocks measured by both flow cytometry and TCID50 assay showed a high level of correlation (Fig. 3, r = 0.930, 2α<0.001).

After elaborating the novel titration assay, we defined its detection limit. A series of 10-fold dilutions of an MV-Schwarz strain stock (6.3 Log IU/ml) was used to evaluate the sensitivity of the technique. To allow the increase of the inoculation volume and to compensate for low viral concentration of some samples, 12-well plates and 4×10^5^ cells were used. Virus concentration, smaller than 4 Log IU/ml, could not be accurately measured by the flow cytometry assay, even when using higher inoculation volumes than 0.3 ml in a final volume of 1 ml of growth medium.

### Titration of MV strains

Wild type MV strains are routinely isolated from infected patients by growing them exclusively on CD150/SLAM expressing cells. In the past, other MV strains have been isolated, passed on Vero cells with acquisition of a tropism for CD46 due to only a few point mutations in the H protein, the most prominent of which is N481Y [20]. These strains are called “laboratory strains” (or Edmonston-like). Some of them have been further attenuated by forced growth in chicken embryonic fibroblasts to give rise to the currently widely used measles vaccine strains. Wild type MV and laboratory/vaccine strains are titrated using Vero-CD150/SLAM, and Vero cells, respectively.

**Figure 4:**
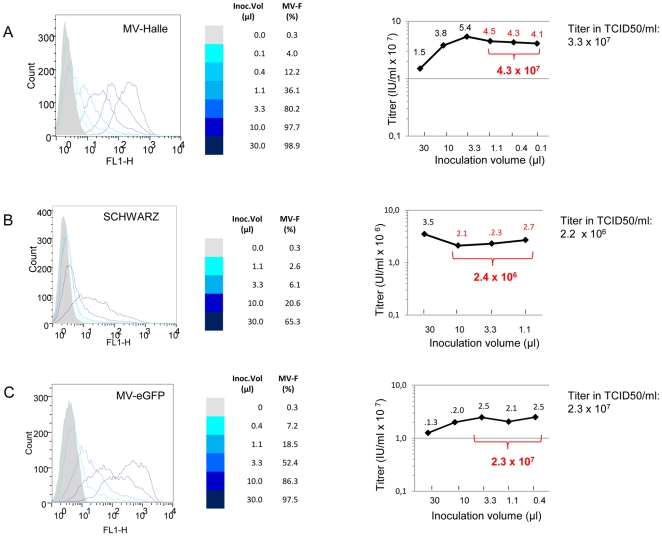
Titer calculation of different measles virus strains. **(A) Calculation of MV-Halle titer based on flow cytometry detection of MV-F protein.** 3-fold serial dilutions of MV-Halle viral stock were used to infect Vero cells. The percentage of infected cells was determined at 18 h.p.i. by flow cytometry. As seen with the histogram, the peak of MV-F positive cells shifts towards the negative cells (left) gradually with the decrease in viral volume used (left panel). The titers expressed as infectious units per milliliter (IU/ml) were calculated for each inoculum size as a percentage of the uninfected cells, according to the Poisson Law (right panel). The values in red were used to calculate the average titer. **(B) Calculation of MV-Schwarz titer based on flow cytometry detection of MV-F protein.** 3-fold dilutions of MV-Schwarz viral stock were used to infect Vero cells. The percentage of infected cells was determined at 18 h.p.i. by flow cytometry (left panel). The titers expressed as infectious units per milliliter (IU/ml) were calculated for each inoculum size as a percentage of the uninfected cells, according to the Poisson's law (right panel). The values in red were used to calculate the average titer. **(C) Calculation of MV-eGFP titer based on the detection of GFP positive cells by flow cytometry.** 3-fold serial dilutions of MV-eGFP viral stock were used to infect Vero cells. The percentage of GFP expressing infected cells was determined at 18 h.p.i. by flow cytometry (left panel). The titers expressed as infectious units per milliliter (IU/ml) were calculated for each inoculum size as a percentage of the uninfected cells, according to the Poisson's law (right panel). The values in red were used to calculate the average titer.

MV laboratory strains (MVHalle, recombinant MV-eGFP, MV-Edmonson), MV vaccine strain (MV-Schwarz), and wild type (MV-G954) virus stocks have been accurately quantified using the rapid method for titration by exactly following the steps from the procedure described in the “Materials and Methods” section above. Examples of MV strains titrations are presented on Figure 4. The titers obtained by the flow cytometry technique were within the error range of those obtained by the TCID50 method.

### The titration by flow cytometry measures only infectious MV particles

Not all viruses produced by the target cells are infectious. Indeed, a large proportion of the virus progeny is defective (non-infectious), and could bind to, and possibly enter into, host cells. Consequently, viral glycoproteins may be found at the cell surface due to the fusion of the viral and cellular membranes leading to an overestimation of the number of infected cells.

To rule out this hypothesis, the rapid titration method was applied to an MV suspension inactivated by UV irradiation at 254 nm for 25 min. Twenty hours post infection and even at the highest virus input (equivalent to MOI = 10), no F protein labeling was detected from the UV inactivated virus while the non-irradiated control virus stock showed the expected titer value of 10^6^ IU/ml (data not shown). Similar results were obtained when MV was heated at 60°C for 20 min, a condition that abolishes virus fusion but not virus binding to the cellular receptor [21]. This indicates that defective virions are not taken into account in the virus titration based on flow cytometry, likely because of the internalization of the adsorbed viral glycoproteins in agreement with the endocytosis of MV bound to its cellular receptor [22].

Thus, the flow cytometry based rapid method measures infectious virus particles (viruses that can undergo at least one complete infection cycle).

### The titration technique by flow cytometry is based on a single round of infection and can be used to determine the duration of the virus replication cycle

The major pitfall that could compromise the accuracy of virus titration by flow cytometry is the overestimation linked to data poisoning by secondary infection. Indeed, during the assay, infected cells progressively become producers of new virions that may spread to cells that have initially escaped infection by the inoculum. Therefore, it is essential to determine the optimal interval separating the virus inoculation and the cell harvest for flow cytometry analysis.

**Figure 5:**
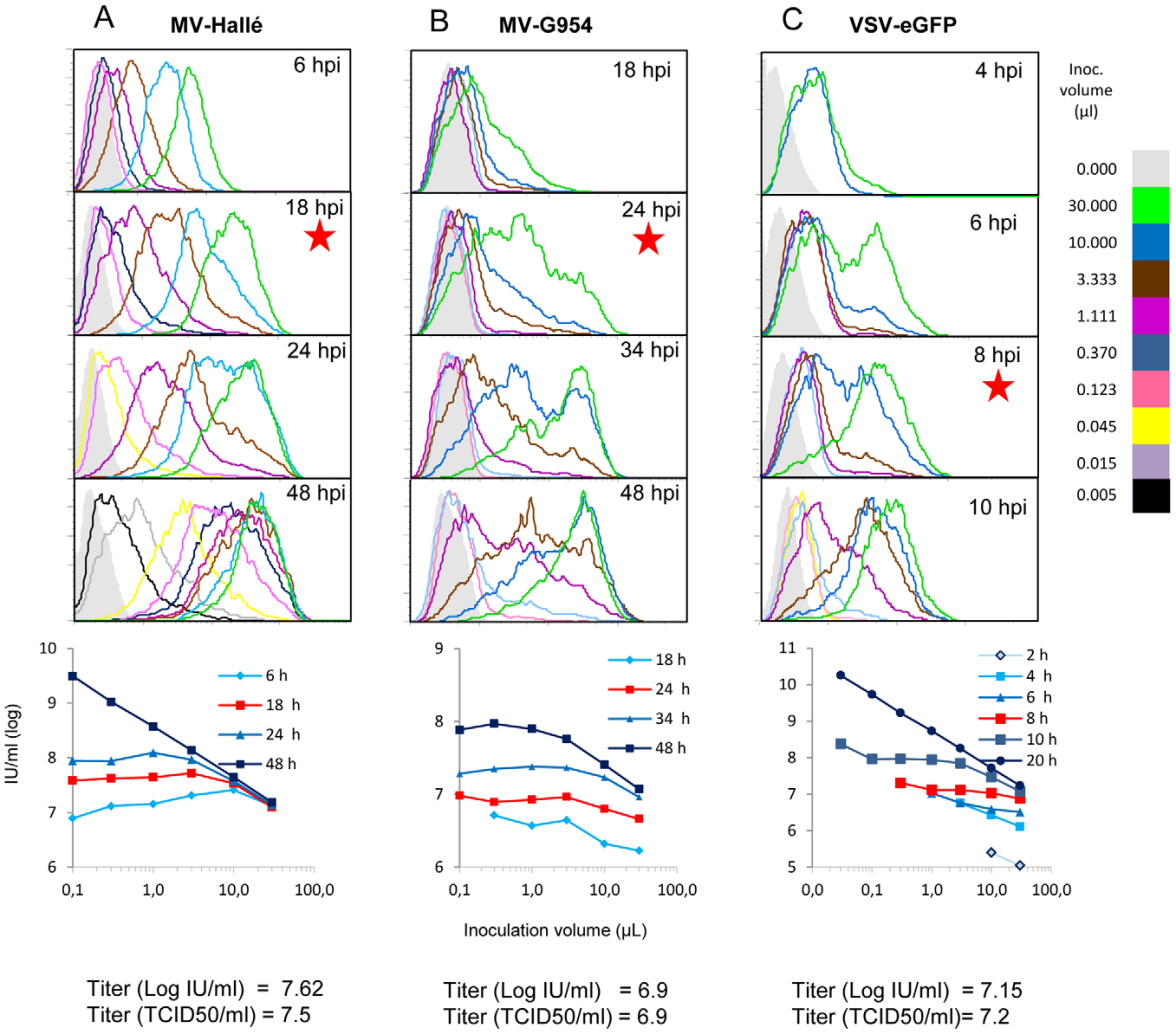
Defining the titration time for different viruses. MV-Halle (panel A), wt MV-G954 (panel B), and VSVeGFP (panel C) stocks were used in 3-fold serial dilutions to infect Vero, Vero-SLAM and Vero cells, respectively. At different times post infection, cells were collected and analyzed by flow cytometry to determine infected cells for each virus input. The optimal time for virus titration reflects the first overlap between the peaks of the most concentrated dilution and the adjacent one and is marked with a “star” on the corresponding histogram. For each dilution, titers have been calculated by applying the Poisson law, and values expressed in IU/ml as function of the inoculation volume (see the charts below the histogram panel for each virus). The red curves reflect the time when a single replication cycle has occurred. The titer is determined by the average of the values on the level curve (red) and is compared to the titer obtained by TCID50 technique below each chart.

This interval could be experimentally determined by examining the histograms of the distribution of MV-F expressing cells after infection by various inoculum volumes over several time points. In the case of MV-Halle strain, the flow cytometry analysis was performed at 6, 18, 24 and 48 hours post infection (h.p.i.) (Fig. 5, panel “A”). The distribution of MV infected cells inoculated with the largest volume (0.03 ml, green histogram) was already close to 100% at 6 h.p.i., but the expression level of F glycoprotein continued to increase up to 18 h.p.i. to remain stable at later time points. This could be interpreted as 18 hours being the time required to reach the maximal expression of F glycoprotein at the surface of an infected cell. When the cells were infected with smaller inoculums, the histograms of infected cells were progressively and regularly shifted to the left. This held true until 18 h.p.i. At 24 h.p.i., the inoculation with the second largest volume (0.010 ml) resulted in a peak shift that partially overlapped with that observed with 0.030 ml (Fig. 5, compare pink and green histograms). At the last time point studied (48 h.p.i.), histograms from the first five virus inputs almost completely overlapped, and even a very small inoculum (MOI as low as 10^−4^) gave rise to a significant percentage of infected cells (black histogram). The time interval allowing maximal expression of the viral gene with the highest inoculum, but not allowing similar expression levels with a lower inoculum size was thus considered to represent the duration of a single virus replication.

For the wild type MV-G954 strain, the optimal incubation time was slightly delayed to 24 h. (Fig. 5, panel “B”).

**Figure 6:**
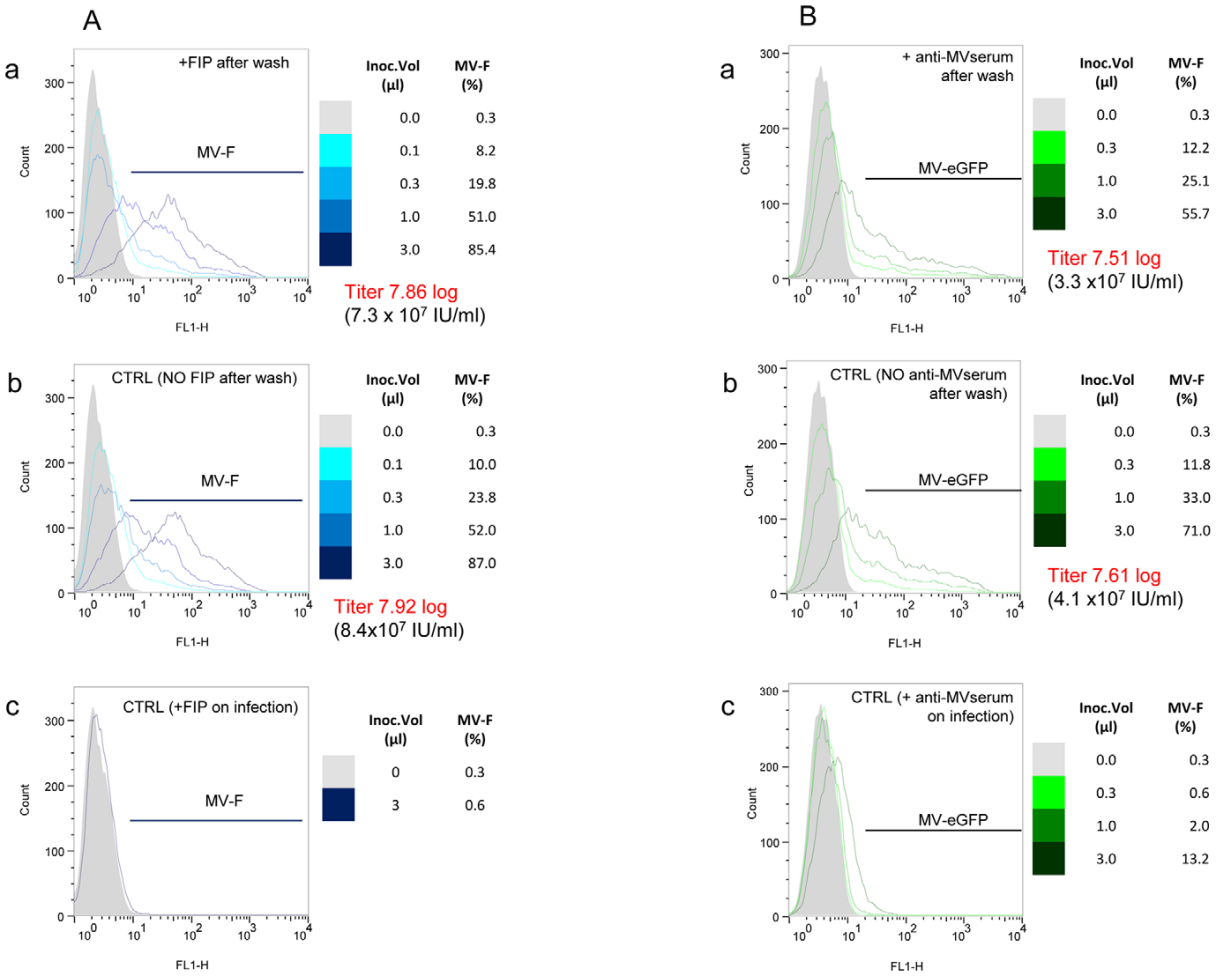
Determination of the duration of a single virus replication cycle and the reading time for titration. **(A) The rapid method for MV titration is based on a single infection cycle.** Vero cells were inoculated with MV-Halle strain for 6 hours. Then, they were washed with PBS to eliminate residual virus. Medium supplemented or not with 0.1 mg/ml of the fusion inhibitory peptide (FIP) was then added. At 18 h.p.i., cells were analyzed by flow cytometry and similar titers were found in both cases (i.e. with and without FIP) (Compare “a” 7.86 Log IU/ml and “b” 7.92 Log IU/ml. As a control for FIP efficiency, its addition just before the virus inoculation completely prevented MV infection “c”. **(B) Validation of estimated virus cycle duration as reading time for titration.** Similar titers were obtained after inhibition of secondary infection by anti-measles serum. Vero cells were infected with MV-eGFP for 6 hours, then washed with PBS and further incubated in the presence “a” or absence “b” of a potent neutralizing antiserum. “c” - neutralization efficiency of the anti-serum upon addition to the virus inoculum prior to infection. Note for the highest inoculum volume, a small GFP background signal was detected due to the low amount of GFP embarked into the input virus particles.

To further confirm the above observations, two complementary experiments using entry inhibitors for *de novo* produced virions were carried out. First, we took advantage of a fusion inhibitory peptide – FIP (z-D-Phe-L-Phe-L-Gly), which above 0.1 mg/ml fully prevents MV entry (Fig. 6A, compare panel “c” with panel “a”). FIP was added at 6 h.p.i. to block any secondary infection. Histogram profiles of MV-Schwarz infected cells observed for each inoculum size at 18 h.p.i. were very similar to the non-treated cultures (Fig. 6, compare panel “b” with panel “a”), thus excluding any detectable contribution of secondary infection when counting the number of infected cells at 18 h.p.i. Second, we added a potent neutralizing antiserum at 6 h.p.i. to prevent secondary infection by MV-eGFP virus. While such treatment almost completely abolished infection, the delayed addition of the antiserum by 6 hours did not interfere with the virus titration (Fig. 6). This strongly suggests that the time interval we have graphically selected as described in Fig. 5 is close to a single virus infection cycle and is optimal to quantify any given infectious virus sample.

Thus, provided that the duration of infection allows only a single cycle of virus replication, the rapid method for measles virus titration is accurate.

### Application for titration of VSV-gfp

The optimal time to titer another virus, VSV-gfp, was determined as 8 h.p.i. (Fig. 5 panel “C”), which fit well with the 6–8 hours duration of the VSV replication cycle [23],[24]. When the apparent titers were blindly calculated for each inoculum and for every time interval, their plotting against the inoculum size gave rise to an almost perfect and expected horizontal distribution (i.e. an identical titer value independent of inoculum size) *only* at the optimal time for each virus (i.e. 18 h.p.i., 24 h.p.i. and 8 h.p.i. red curves, bottom graphs). Finally, the titer, determined by the rapid titration procedure at 8 h.p.i. correlated best with the titer obtained by the classical TCID50 method.

### Application for titration of HIV-1

**Figure 7:**
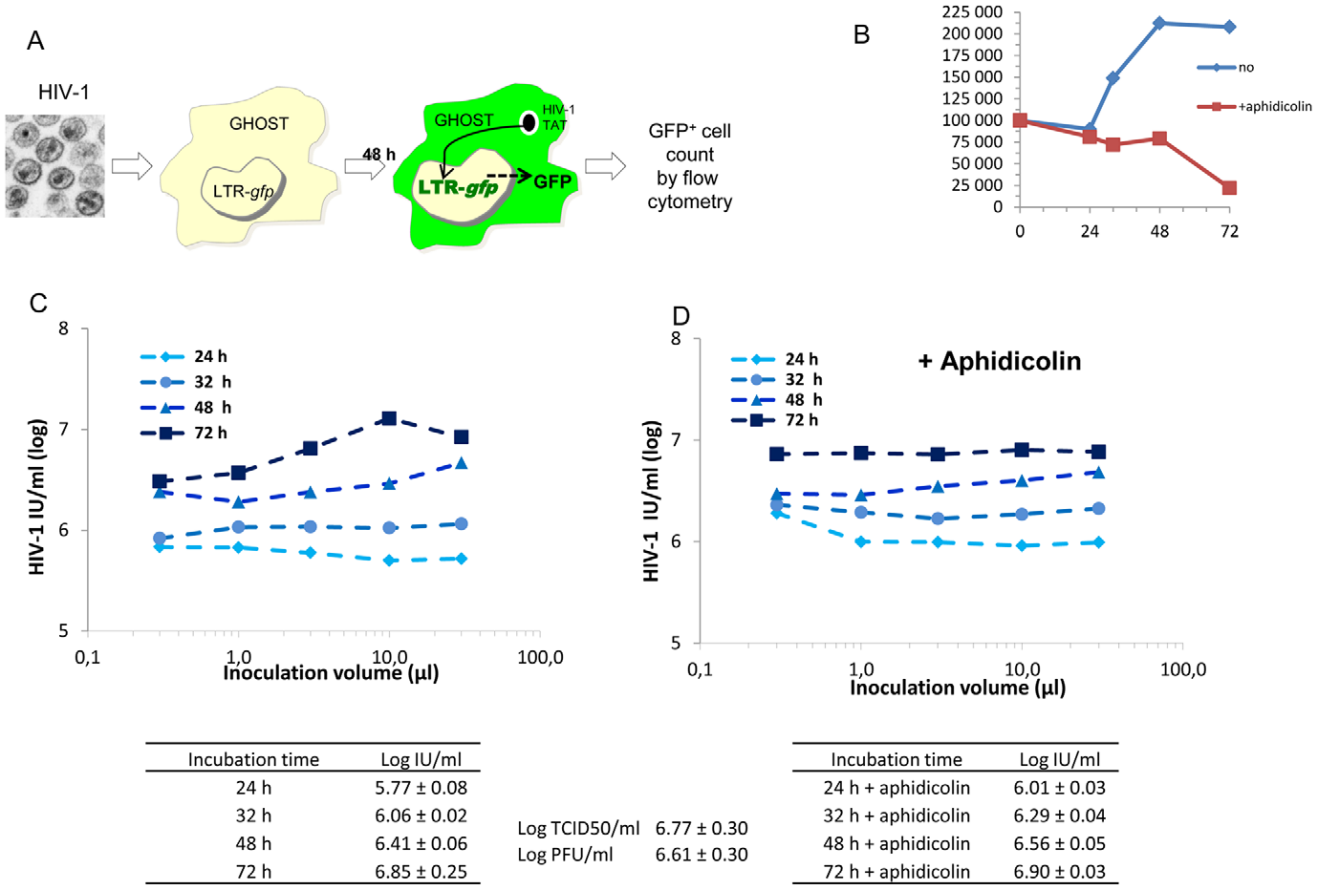
Calculation of HIV-1 NL4-3 titer using GFP expressing indicator cells. HIV-1 (NL4-3 strain) stock was titrated both by serial dilutions in SupT1 cells and GHOST cells. End-point titration using the lymphocytic cell line SupT1 (data not shown) was based on the appearance of syncytia and took up to 2 weeks. The GHOST indicator cells were used for HIV-1 titration by flow cytometry. These cells express the GFP protein under the control of the HIV-1 LTR that can be activated by Tat from the incoming HIV-1 (A). GHOST cells seeded one hour before were infected with serial 3-fold dilutions of HIV-1 stock in the absence (C) or presence (D) of 1 µg/ml aphidicolin. At various times post-infection, cells were collected, fixed and GFP expression analyzed by flow cytometry. In parallel, at every time point, the cells were collected from non-infected wells to count the cell number for cell growth determination (B).

The protocol was adapted to quantify HIV-1 using indicator cells expressing a reporter gene product upon activation from an incoming virus. GHOST HIV-1 indicator cells encode for a GFP protein under the control of HIV-1 LTR and thus respond to Tat protein expression resulting from an HIV-1 infection by becoming fluorescent (Fig. 7A) [25]. As HIV-1 infection is rather slow, a kinetics experiment was done over 72 hours. To overcome any interference of cell number changes due to proliferation during the titration assay, the experiment was duplicated in the presence of aphidicolin, a reversible inhibitor of the DNA polymerase. Indeed, after one day lag period, cells started to proliferate to double in number within two days of infection (Fig. 7B) and stop growing thereafter. The aphidicolin treatment prevented any increase in cell number without affecting cell viability over the first 48 h. Virus titration over a wide range of inoculation volumes was found to be more reliable in the presence than in the absence of aphidicolin (compare Fig. 7C with Fig. 7D online), and titers determined after 48 h of culture were within the error range of that determined by the TCID50 assay.

### Application for titration of HIV-1 based lentivectors

Different virus inputs (10 µl, 3 µl, 1 µl, 0.3 µl) from a concentrated stock of HIV-1 based lentiviral particles (pseudotyped with VSV envelope G protein and expressing GFP as a transgene [26]–[28]) were used to infect Vero cells according to the procedure already described above. 24 hours afterwards, the cells were trypsinized and GFP positive cells were measured by flow cytometry. The titer was calculated for each input, and values below 50% of F^+^ cells were averaged to calculate the titer. Example: for virus volumes 0.3 µl, 1 µl, 3 µl and 10 µl the percentage of infected cells was 15.3%, 43.7%, 72.4% and 96.3%, respectively. Therefore the virus titer (as average) was calculated from the 2 lowest flow cytometry values and was 7.75 Log IU/ml (which corresponds to (7.74 Log IU/ml for 15.3%+7.76 Log IU/ml for 43.7%)/2 = 7.75).

### Comparison with other available virus titration methods

In comparison with other flow-cytometry based methods described previously to titer different viruses [3]–[13], our technique appears original and complementary in several aspects: (i) a complete set of critical parameters has been fully explored and experimentally validated; (ii) our method is the first one to describe a flow-cytometry based procedure to determine the duration of a single virus replication cycle, a key parameter for titration accuracy, and (iii) the first rapid method for quantifying different measles virus strains; (iv) adjustments of critical parameters allowed its use to titer two other unrelated viruses, VSV and HIV-1; (v) by applying the Poisson law the titer calculation appeared to be very precise and highly reproducible.

Compared to the PFU method and TCID50, the flow cytometry based method has the advantage of being rapid which might be crucial in vaccine production and when short deadlines in research laboratories should be met. Moreover, it could be used to titer non-replicative virus particles (e.g. lentiviral vectors), and a large number of virus samples may be compared simultaneously thanks to limited handling and high precision. However, there are two major limitations: (i) the detection limit is 10^4^ IU/ml, which prevents its use for titration of clinical samples that often have a low virus load, and (ii) it is not recommended for viruses (mutants, etc.) with replication cycles exceeding more than 2 days. Alternative methods have been designed based on the measure of either the genome content of a viral suspension [12] or cell-associated viral nucleic acids by quantitative real-time PCR after end point dilution of the infecting virus (PCR infectivity assay) [29]–[30]. However, the obtained viral nucleic acid values have to be calibrated using an infectious virus reference, and any change in the [nucleic acid/infectious unit] ratio prevents the accurate determination of the number of infectious particles. The advantages/disadvantages and specific required equipment of every titration methods have been listed in Table 2 as a helpful tool to choose the most suitable titration procedure.

### Conclusion

The virus titration method based on the detection of infected cells by flow cytometry after a time interval corresponding to one virus replication cycle proved to be reproducible, accurate, rapid (one virus replication length), and with a detection limit of 10^4^ IU/ml. Furthermore, it could be easily automated [31]. Titers obtained through this technique correlate significantly with those determined by the classical TCID50 method but with higher accuracy and a 3-fold lower range of confidence limit. Such a method may be particularly beneficial for studies requiring titration of large numbers of MV stocks and especially when strict deadlines should be respected. In principle this method can be adapted to titer any virus for which a viral protein can be easily detected by flow cytometry, including recombinant viruses carrying a fluorescent reporter gene (or any gene encoding a cell surface protein that could be immunolabelled) and the use of indicator cells with an endogenous reporter gene that is inducible upon virus infection (see Table 1 for setting up steps of a new virus titration assay). The method is not suitable for titration of viruses (e.g. recombinant viruses, mutants, etc.) which replication cycle takes more than 30 h unless the cell proliferation is prevented. Indeed, the accuracy of virus titration critically relies on the precision of seeded cell number that is used in the formulae to calculate the titer. Any significant cell growth during the assay may result in the underestimation virus titer because infected cells usually grow slower than uninfected cells. This method could prove useful to titrate HIV and HIV molecular clones designed to undergo a single viral cycle. Moreover, this method also allows determination of the duration of a virus replication cycle, a key parameter for the accuracy of the virus titration.

**Table 1:** Adaptation of the titration protocol for a given virus.

**I Choice of cell line**	Use a highly permissive cell line on which the virus can be isolated and produced.For lentiviral vectors pseudotyped with VSV G protein, chose the cell line that you most commonly use for your experiments.
**IIa Labelling: choice of the virus encoded marker detectable by flow cytometry**	**For enveloped viruses:** use cell surface glycoprotein if well expressed and good antibodies are available.**For non-enveloped viruses:** use intracellular immunolabelling against a structural viral protein. In that case, a permeabilisation step prior antibody labelling is necessary.**For GFP expressing viruses:** follow GFP expression.**For lentiviral vectors:** use appropriate labelling against the transgene (if the transgene is not a fluorescent protein).
**IIb Labelling: optimization**	Optimized the immunolabelling conditions (antibody choice, saturating concentration of immunoreagents, choice of permeabilization procedure that minimize non-specific labelling) on cells infected at a MOI of 0.1 to 1.
**III Determination of the duration of the virus replication cycle**	1. Perform infection kinetics using 3-fold dilutions of the virus stock for different time points (i.e. 6 h, 8 h, 18 h, 24 h, 30 h, 36 h, and 48 h).2. Calculate titres for each dilution used and plot them on a graph «titer/inoculation volume» for each time point. The average titre obtained from all dilutions per time point should be noted (preferably, MOIs between 0,5 and 0,02 should be considered).3. Perform titration of your viral stock using an already described and validated method, i.e. TCID50. Note the titre.4. Define the single replication cycle by analyzing the flow cytometry histograms: note the time point where the peak of the second virus input starts to approach and overlap the peak of the first (highest) virus input)5. Correlate the data from point 2, 3 and 4 above. The duration of the replication cycle should correspond to the time point where the slope of the time course is close to 0. If more than one similar slope is available, choose the one which gives the titre that correlates best with the titre in TCID50.6. Choose the length of the virus replication cycle as the infection duration for the titration procedure.
**IV Confirmation**	Check that the time interval define in III is correct by comparing the virus titres obtained in the absence and in the presence of either an antibody (serum), or a virus entry inhibitors that block secondary infection added few hours after the virus inoculation (see text for details).
**V Exclude false positive signal**	Inactivate your virus by UV treatment and check if you still get labelling by flow cytometry. If yes, virus titration by flow cytometry is not recommended.
**VI Validation**	Perform multiple titration of the same viral stocks using an already accepted technique i.e. TCID50/PFU and the novel method.
**VII Detection limit**	Prepare 10-fold (or 5-fold) dilutions of your viral stock and titre each of them.

**Table 2:** The advantages and disadvantages of existing methods to titrate viruses.

	Plaque (PFU)	TCID50	qRT-PCR	qPCR infectivity	Flow cytometry
**Duration**	5–9 days	5–12 days	hours	1 day	1 day
**Sensitivity^1^**	1/ml	100/ml	(1/ml ?)^2^	(100/ml ?)^2^	10^4^/ml
**Type of measurement**	continuous	discontinuous	continuous	continuous	continuous
**Measure**	Infectivity, absolute	Infectivity, absolute	Genome relative	Genome relative	Infectivity, absolute
**Need of a Reference**	no	no	yes	yes	no
**Specific Equipment**	-	-	RT-PCR(expensive apparatus and reagents)	RT-PCR(expensive apparatus and reagents)	Flow cytometer(expensive apparatus, cheap reagents)
**Specific reagents**	-	-	Primers to be designed	Primers to be designed	Specific Antibody against a major viral protein or a fluorescent reporter gene
**Costs^1^**	medium	low	high	high	medium
**Human labor**	high	high	medium	medium	low
**Disadvantages**	CumbersomeTime consumingOperator dependentSubjective CountingHigh STDEV^4^	CumbersomeTime consumingImportant STDEV – 0.3 Log	Do not measure infectivity	Valid only when genome to IU is invariantReplication rate dependent	Limited sensitivityReplication rate dependentNot suitable if virus replication cycle >2–3 days
**Advantages**	Very high sensitivity	High sensitivity	Rapid	Rapid	RapidVery low STDEV<0.1 Log
**Prerequisites**	none	none	Primer choiceqPCR settingCalibration to IU	Primer choiceqPCR settingCalibration to IU	Duration of a single virus replication cycle to be determined first
**Suitability**					
**Determination of virus cycle duration**	no	no	no	no	yes
**Detection/Titration of infectious virus in clinical samples**	Excellent	Good (titer>100 IU)	no	Good (titer>100 IU) ?	no
**Restricted to viruses able to induce a CPE**	yes	no	no	no	no
**VIRUS TITRATION**	Good	Very Good	no	Good/very Good	Excellent
**Titration of lentiviral vectors (non-replicative)**	no	no	no	no	Excellent
**Multiple sample titration**	Limited by high human labor	Good	Good	Good	Excellent
